# Selenomethionine alleviates LPS-induced septic kidney injury by regulating mitochondrial dynamics changes

**DOI:** 10.3389/fphar.2025.1606365

**Published:** 2025-09-08

**Authors:** Xu Zhou, Jiling Zhao, Wusong Cheng, Su Chen, Ya Zhang, Yangang Qu, Xiaohui Hu, Yong Lan, Qimin Tu, Jiaxin Hu, Hongbo Chen

**Affiliations:** ^1^ Department of Urology, Central Hospital of Enshi Tujia and Miao Autonomous Prefecture, Enshi, China; ^2^ Hubei Selenium and Human Health Institute, The Central Hospital of Enshi Tujia and Miao Autonomous Prefecture, Enshi, Hubei, China; ^3^ Cardiovascular Center, Central Hospital of Enshi Tujia and Miao Autonomous Prefecture, Enshi, China; ^4^ Department of Obstetrics and Gynecology, Enshi Maternal and Child Health Hospital, Enshi, China; ^5^ Department of Pathology, Central Hospital of Enshi Tujia and Miao Autonomous Prefecture, Enshi, China; ^6^ Cardiothoracic Surgery, Central Hospital of Enshi Tujia and Miao Autonomous Prefecture, Enshi, China

**Keywords:** acute kidney injury, lipopolysaccharide, macrophages, mitochondria, NF-κB

## Abstract

**Background:**

Acute kidney injury (AKI) is a prevalent complication of sepsis, where the inflammatory response plays a crucial role. Selenium exhibits anti-inflammatory and antioxidant properties, but its impact on sepsis-induced AKI remains unclear.

**Methods and results:**

In this study, we used a lipopolysaccharide (LPS)-induced murine model of sepsis-associated acute kidney injury (SA-AKI) in male C57BL/6 mice (8–12 weeks old) to investigate the protective mechanisms of selenomethionine (SeMet). Mice received weekly oral administration of SeMet (0.375 mg/kg) commencing 1 week prior to AKI induction. Our results demonstrated that SeMet treatment significantly attenuated the inflammatory response, reduced oxidative stress, and ameliorated renal pathological damage compared to saline-treated controls. Mechanistic investigations revealed that SeMet modulates altered mitochondrial dynamics and suppresses the NF-κB signaling pathway, thereby promoting macrophage polarization toward the anti-inflammatory M2 phenotype.

**Conclusion:**

These findings collectively demonstrate that SeMet effectively mitigates inflammation and ameliorates sepsis-induced AKI, suggesting its potential as a therapeutic candidate for SA-AKI prevention and treatment.

## 1 Introduction

Sepsis, characterized as a systemic inflammatory response syndrome, frequently results in multi-organ dysfunction (Gotts and Matthay, 2016). Sepsis-associated acute kidney injury (SA-AKI) involves progressive renal dysfunction triggered by sepsis or septic shock (Qiao et al., 2024), contributing to high in-hospital mortality (>25% in severe AKI and >50% with renal replacement therapy) (Legrand et al., 2024). Consequently, the identification of effective therapeutic agents to improve outcomes in SA-AKI patients represents a critical clinical imperative.

Macrophages (Mϕs) demonstrate remarkable plasticity, capable of adopting diverse functional phenotypes in response to various stimuli, thereby playing pivotal roles in regulating inflammatory responses during sepsis (Chen et al., 2021). The macrophage population primarily comprises two distinct phenotypes: classically activated M1 macrophages and alternatively activated M2 macrophages. M1 macrophages secrete numerous pro-inflammatory mediators, including IL-1, TNF-α, and IL-6, which participate in host defense against pathogens (Murray and Wynn, 2011). In contrast, M2 macrophages release anti-inflammatory mediators such as IL-10 and TGF-β, which facilitate tissue repair (Chen et al., 2019), and express immunomodulatory proteins including Fizz1 and Arg1 that have been demonstrated to attenuate inflammation and promote wound healing (Wang and Harris, 2011). During systemic inflammatory responses, aberrant macrophage recruitment and persistent activation can ultimately precipitate acute organ dysfunction. Emerging evidence suggests that promoting macrophage polarization toward the M2 phenotype may exert potent anti-inflammatory effects (Peng et al., 2024).

Mitochondrial dynamics encompass the continuous processes of fission and fusion in these highly dynamic organelles, profoundly influencing mitochondrial reactive oxygen species (ROS) production, calcium homeostasis, and oxidative phosphorylation (Chan, 2020). The regulation of mitochondrial mass primarily depends on the balance between fusion and fission processes, mediated by specific proteins, including Drp1, Opa1, and Fis1 for fission, and Mfn1 and Mfn2 for fusion (Otera et al., 2013). The interplay between fission and fusion facilitates efficient mitochondrial trafficking, optimizes oxidative phosphorylation (OXPHOS), and enhances mitochondrial mass homogenization, thereby enabling redistribution of mitochondrial DNA (mtDNA) between compromised and healthy mitochondria (Chan, 2020). Notably, studies have established that OXPHOS provides essential energy for M2 macrophage polarization (Bailey et al., 2019). Perturbations in mitochondrial dynamics can impair OXPHOS capacity, consequently affecting macrophage polarization toward the M2 phenotype. Furthermore, alterations in Drp1 and Mfn1 expressions can influence NF-κB signaling pathway activity and ROS generation (Huang et al., 2016), providing mechanistic insights relevant to the current investigation.

Selenium (Se), an essential micronutrient, plays critical roles in development and various physiological processes. As an organic selenium compound, selenomethionine (SeMet) demonstrates superior efficacy in elevating selenium concentrations in biological systems compared to other selenium compounds (Zhuang et al., 2020). SeMet exhibits diverse biological functions, including antioxidant, anti-inflammatory, immunomodulatory, and metabolic regulatory activities (Wang Y. et al., 2024; Huang et al., 2012). Previous research has shown that SeMet can restore mitochondrial kinetic stability (Chen X. et al., 2024) and facilitate macrophage transition from pro-inflammatory M1 to anti-inflammatory M2 phenotypes (Nelson et al., 2011). Given the established contribution of inflammatory responses to SA-AKI progression (Xia et al., 2019), elucidating the effects of SeMet on inflammatory mediators assumes particular significance. This investigation aimed to evaluate the regulatory effects of SeMet on macrophage polarization and its protective benefits in SA-AKI. Our findings reveal that SeMet exerts significant anti-inflammatory effects, effectively mitigating SA-AKI progression by promoting M2 macrophage polarization. This immunomodulatory effect appears mediated through the regulation of mitochondrial dynamics and suppression of the NF-κB signaling pathway. These discoveries provide promising insights for developing targeted therapies for clinical SA-AKI management.

## 2 Materials and methods

### 2.1 Cell culture

RAW264.7 murine macrophage cells were procured from Wuhan Sunncell Biotech Co., Ltd. (China) and maintained in high-glucose Dulbecco’s modified Eagle medium (DMEM) supplemented with 10% fetal bovine serum (Nanjing BioChannel Biotechnology Co., Ltd., China, Cat: BC-SE-FBS08) and antibiotic–antimycotic solution (100 U/mL penicillin and 100 μg/mL streptomycin; Thermo Fisher Scientific, USA, Cat: 11995500BT). Cells were cultured at 37 °C in a humidified incubator with 5% CO_2_. For experimental procedures, cells were allocated into four treatment groups: (1) control group (medium only), (2) SeMet group [1 μM SeMet for 24 h, based on previous studies (Shen et al., 2015)], (3) LPS group (1 μg/mL LPS for 24 h), and (4) LPS + SeMet group (co-treatment with 1 μg/mL LPS and 1 μM SeMet for 24 h).

### 2.2 Animal experiments

Male C57BL/6 wild-type mice (6–10 weeks old) were obtained from Wuhan Hualianke Biotechnology Co., Ltd. (China) and housed under specific pathogen-free conditions with *ad libitum* access to food and water. Environmental parameters included a 12-h light/dark cycle, ambient temperature maintained at 25 °C ± 1 °C, and a relative humidity of 55% ± 5%. All animal procedures received approval from the Institutional Animal Care and Use Committee at the Central Hospital of Enshi Tujia and Miao Autonomous Prefecture (Approval No: 202403003). Mice were randomly divided into four experimental groups (*n* = 10 per group): (1) control, (2) LPS group (intraperitoneal injection of 10 mg/kg LPS), (3) SeMet group, and (4) SeMet + LPS group (oral gavage of 0.375 mg/kg SeMet administered 1 day prior to LPS injection). Survival rates were monitored for 1 week following septic AKI model induction. Subsequently, the SA-AKI model was re-established with six mice per group using identical treatment protocols. All animals were euthanized 24 h post-LPS administration, followed by immediate collection of kidney tissues and cardiac puncture blood samples. SeMet (Cat: HY-B1000) and LPS (Cat: HY-D1056) were purchased from MedChemExpress (USA).

### 2.3 Cell viability assay

Cell viability was assessed using the Cell Counting Kit-8 (CCK-8; Shanghai Beyotime Biotechnology Co., Ltd., China; Cat. No.: C0038). In brief, RAW264.7 cells were seeded in 96-well plates at a density of 1 × 10^4^ cells/well in 200 μL complete medium. Upon reaching 40%–50% confluence, cells were treated with SeMet at concentrations ranging from 0.1 to 100 μM for 24 h before CCK-8 assay implementation.

### 2.4 Real-time quantitative reverse-transcription PCR (qPCR)

Total RNA extraction was performed using a rapid RNA isolation kit (Aidlab Biotechnologies Co., Ltd., China; Cat. RN07), followed by cDNA synthesis using a reverse transcription kit (Shanghai Tolo Biotechnology Co., Ltd., China; Cat. 22107). Quantitative PCR analysis was performed using a SYBR Green-based qPCR kit (Shanghai Tolo Biotechnology Co., Ltd., China; Cat. 22204) on a real-time PCR system. GAPDH served as the endogenous reference gene for normalization. Primer sequences are provided in Supplementary Table S1.

### 2.5 Western blotting

Proteins were isolated using RIPA buffer (Abbkine, Cat# BMP1001), separated using SDS-PAGE, and transferred to PVDF membranes (Millipore, Cat# IPVH00010, Burlington, MA). Membranes were probed overnight (4 °C) with primary antibodies (all ABclonal): β-actin (AC026), Arg1 (RP00247), iNOS (A3774), Mfn1 (A9880), Mfn2 (A19678), Drp1 (A21968), Opa1 (A9833), NF-κB (A19653), IκBα (A19714), p-IκBα (AP0707), p-NF-κB (AP355), IKKα (A19694), and p-IKKα (AP0506) (1:500–1:1000). Secondary antibodies were applied (2 h, RT). Protein levels were quantified using Image Pro-Plus. Membranes were stripped (ServiceBio, Cat# G2079-100 ML, Wuhan, China) for re-probing.

### 2.6 Mitochondrial membrane potential assessment (ΔΨm)

ΔΨm was evaluated using the JC-1 Assay Kit (Shanghai Beyotime Biotechnology Co., Ltd., China; Cat: C2006) according to the manufacturer’s instructions. In healthy mitochondria with intact membrane potential, JC-1 forms red-fluorescent J-aggregates, whereas depolarized mitochondria retain green-fluorescent JC-1 monomers. Fluorescence was visualized using an inverted fluorescence microscope (Olympus IX71) at ×20 magnification.

### 2.7 Measurement of lactate (LA) and ATP

Lactate and ATP concentrations were determined using commercial assay kits (Beijing Solarbio Science & Technology Co., Ltd., China; LA: Cat: BC2235; ATP: Cat: S0026), following the manufacturer’s protocols.

### 2.8 H&E staining

Kidney tissues were fixed in 4% paraformaldehyde, paraffin-embedded, and sectioned at 4 μm thickness for hematoxylin and eosin (H&E) staining. Pathological evaluation was performed by light microscopy.

### 2.9 TUNEL staining

Apoptosis was assessed using a TUNEL kit (Shanghai Beyotime Biotechnology Co., Ltd., China, Cat: C1098) on 5 μm-thick paraffin-embedded kidney sections according to the manufacturer’s instructions.

### 2.10 Immunofluorescence staining

Cells were fixed with 4% paraformaldehyde, permeabilized with 0.25% Triton X-100 (Jiangsu Labgic Technology Co., Ltd., Cat: BS084), blocked with BSA, and incubated with primary antibodies overnight at 4 °C followed by appropriate secondary antibodies. Nuclei were counterstained with DAPI. Images were acquired using a confocal microscope (Leica TCS SP8) with a ×200 objective.

### 2.11 Transmission electron microscopy (TEM)

Kidney tissues were fixed in 4% glutaraldehyde at 4 °C for 24 h, post-fixed in osmium tetroxide, dehydrated through a graded ethanol series, and embedded in resin. Before examination, ultrathin sections (50–60 nm) were stained with uranyl acetate and lead citrate using a transmission electron microscope (HT7700, Hitachi, Japan). For cell samples, cultured macrophages were similarly processed and examined using an FEI Tecnai 12 microscope (Philips, Netherlands).

### 2.12 Statistical analysis

Data are presented as mean ± standard error of the mean (SEM) from at least three independent experiments. Intergroup comparisons were performed using Student’s t-test for two groups or one-way ANOVA followed by Tukey’s *post hoc* test for multiple groups. All statistical analyses were conducted using GraphPad Prism 5.0 (GraphPad Software, USA), with p < 0.05 considered statistically significant.

## 3 Results

### 3.1 SeMet attenuates renal pathological damage and improves survival in SA-AKI mice

**FIGURE 1:**
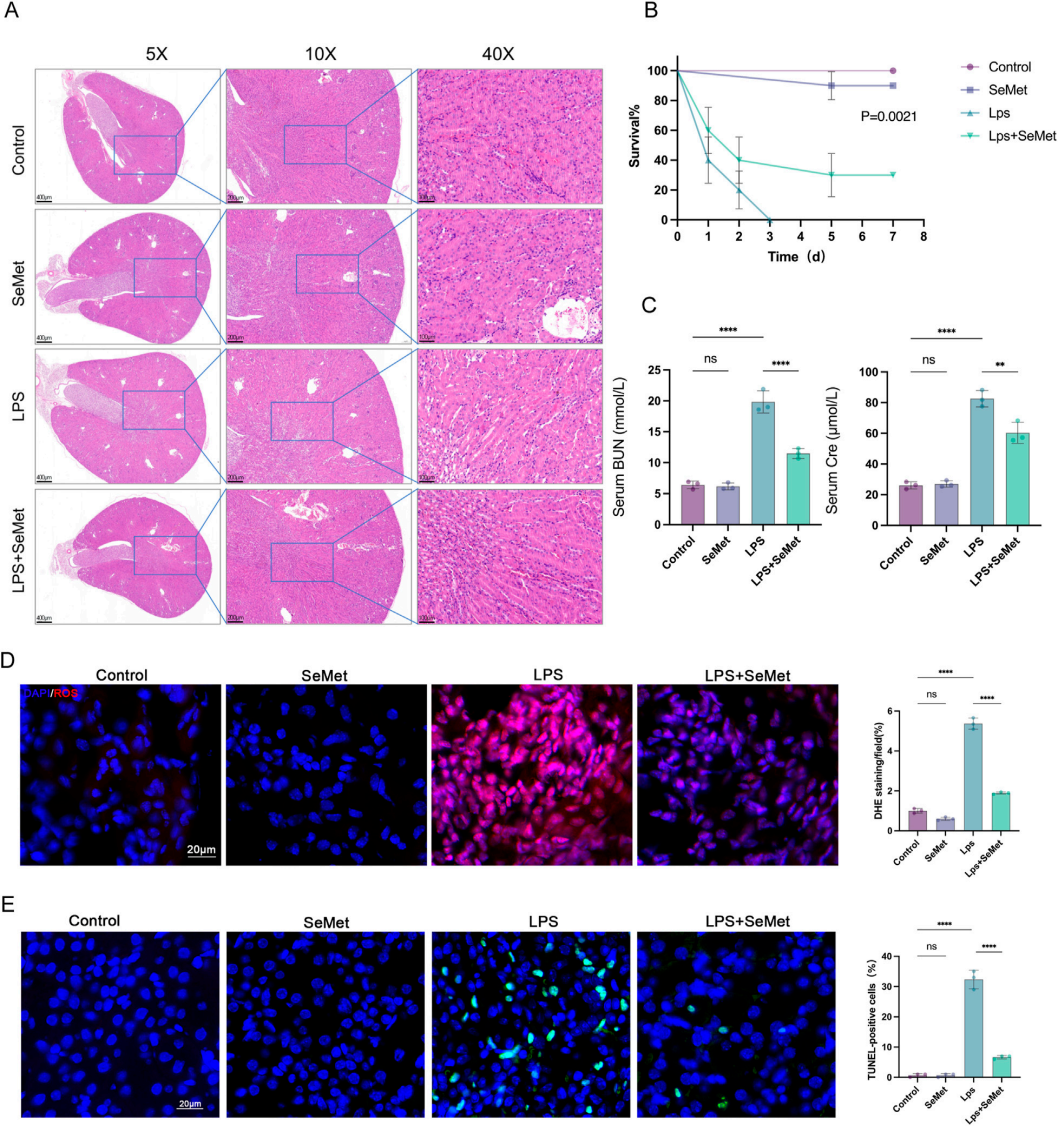
SeMet improves kidney pathological changes and reduces mortality in SA-AKI mice. **(A)** H&E staining of kidney tissue sections from control, SeMet, LPS, and LPS + SeMet groups, with magnifications of ×5, ×10, and ×40 (from left to right) (scale bars from left to right: 400 μm, 200 μm, and 100 μm) (*n* = 3). **(B)** Seven-day survival curves in mice of the control, SeMet, LPS, and LPS + SeMet groups. **(C)** Serum BUN and creatinine levels in mice of the control, SeMet, LPS, and LPS + SeMet groups (*n* = 3). **(D)** Representative immunofluorescence images and fluorescence quantification of ROS in control, SeMet, LPS, and LPS + SeMet mice (*n* = 3), with staining for DAPI (blue) and ROS (red) (scale bar, 20 μm). **(E)** Representative immunofluorescence images and fluorescence quantification of TUNEL in control, SeMet, LPS, and LPS + SeMet mice (*n* = 3), with staining for DAPI (blue) and TUNEL (green) (scale bar, 20 μm).

To elucidate the protective role of SeMet in SA-AKI, we established an LPS-induced kidney injury model in C57BL/6J mice. Histopathological examination revealed that SeMet intervention significantly ameliorated LPS-induced proximal tubular dilatation, interstitial widening, and tubular necrosis compared to untreated controls (Figure 1A). Importantly, SeMet pretreatment markedly improved survival rates in septic mice (Figure 1B). Biochemical analyses demonstrated that SeMet treatment effectively reversed LPS-induced elevations in serum urea nitrogen and creatinine levels (Figure 1C). Given the established antioxidant and antiapoptotic properties of SeMet, we further evaluated renal oxidative stress and apoptosis. Our results showed significant reductions in both ROS levels and TUNEL-positive apoptotic cells in SeMet-treated animals compared to LPS-only controls (Figures 1D,E). These findings collectively demonstrate that SeMet confers substantial protection against sepsis-induced renal injury.

### 3.2 SeMet modulates macrophage polarization and attenuates systemic inflammation

**FIGURE 2:**
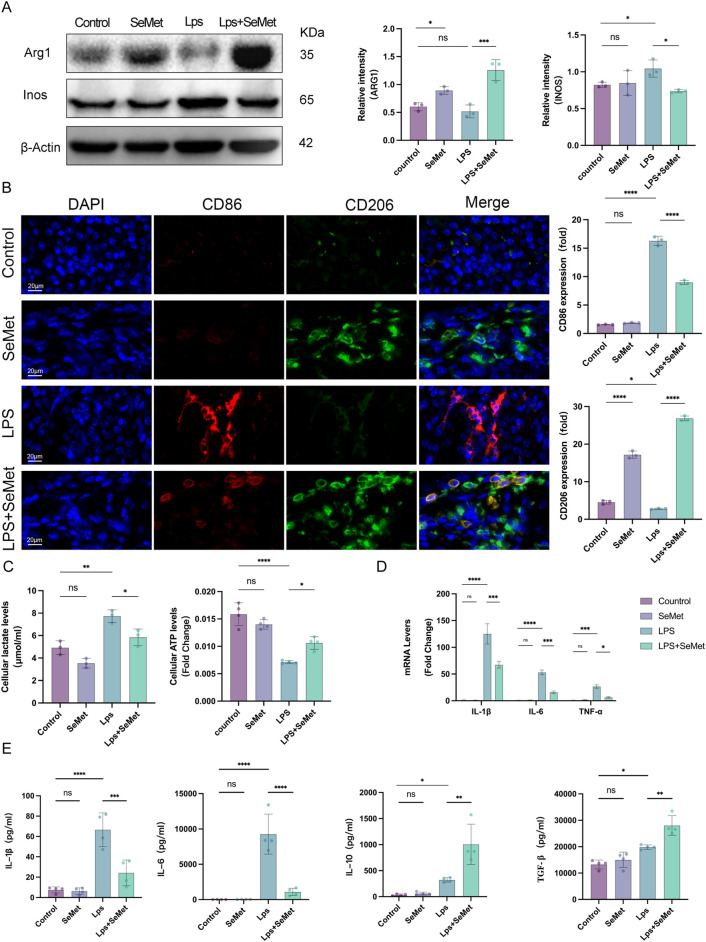
SeMet induces macrophage polarization toward the M2 phenotype and alleviates the inflammatory response in septic mice. **(A)** Representative Western blots of ARG1 and INOS, along with densitometric analysis, in mice of the control, SeMet, LPS, and LPS + SeMet groups (*n* = 3). **(B)** Representative immunofluorescence images and fluorescence quantification of CD86 and CD206 in mice of the control, SeMet, LPS, and LPS + SeMet groups (*n* = 3), with staining for DAPI (blue), CD86 (red), and CD206 (green) (scale bar, 20 μm). **(C)** Levels of ATP and lactate in kidney tissues of mice treated with control, SeMet, LPS, and LPS + SeMet (*n* = 3). **(D)** RT-qPCR analysis of IL-1β, IL-6, and TNF-α expressions in mice of the control, SeMet, LPS, and LPS + SeMet groups (*n* = 3). **(E)** ELISA measurement of serum levels of IL-1β, IL-6, IL-10, and TGF-β in control, SeMet, LPS, and LPS + SeMet groups (*n* = 3).

As macrophage polarization plays a pivotal role in inflammatory regulation, we examined polarization markers in renal tissues. Western blot analysis revealed significantly increased Arg1 (M2 marker) expression and decreased iNOS (M1 marker) levels in SeMet-treated groups compared to LPS controls (Figure 2A). Immunofluorescence staining confirmed these findings, showing enhanced CD206 (M2 marker) expression and reduced CD86 (M1 marker) expression in SeMet-treated kidneys (Figure 2B). Metabolic analyses demonstrated that SeMet treatment normalized LPS-induced metabolic disturbances, reducing lactate accumulation while restoring ATP production (Figure 2C). At the molecular level, SeMet significantly downregulated pro-inflammatory cytokines (IL-1β, IL-6, and TNF-α) while upregulating anti-inflammatory mediators (IL-10 and TGF-β) at both mRNA and protein levels (Figures 2D,E). These results indicate that SeMet promotes an anti-inflammatory microenvironment through macrophage polarization.

### 3.3 SeMet enhances mitochondrial fusion and promotes M2 polarization in macrophages

**FIGURE 3:**
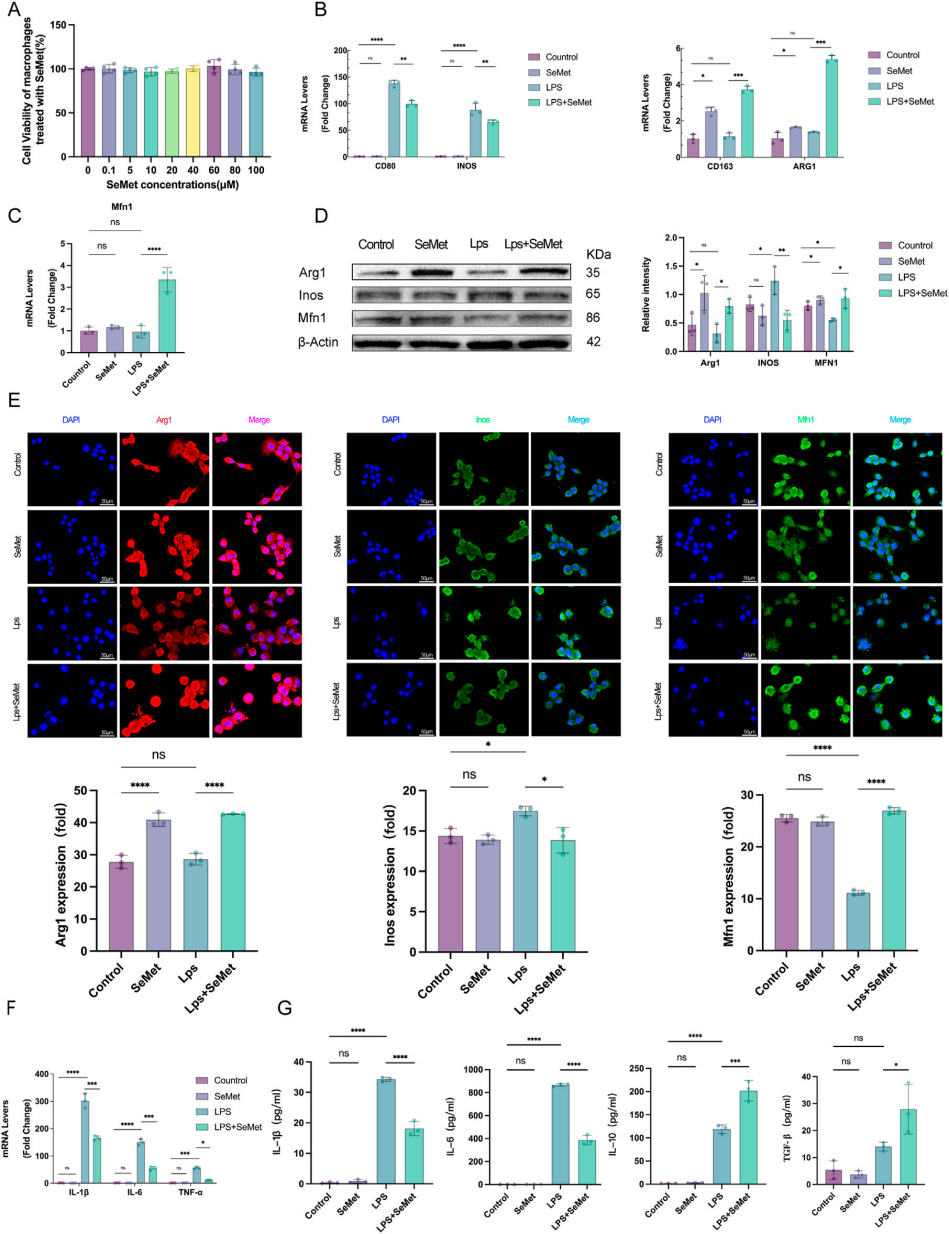
SeMet-induced mitochondrial fusion promotes M2 polarization in RAW264.7 cells. **(A)** Cell viability was assessed using CCK-8 to evaluate the cytotoxicity of SeMet (0.1, 5, 10, 20, 40, 60, 80, and 100 μM) (*n* = 5). **(B,C)** RT-PCR analysis of mRNA levels of CD80, CD86, INOS, CD163, ARG1, and MFN1 in mice of the control, SeMet, LPS, and LPS + SeMet groups. **(D)** Representative Western blots of ARG1, INOS, and MFN1, along with densitometric analysis, in mice of the control, SeMet, LPS, and LPS + SeMet groups (*n* = 3). **(E)** Representative immunofluorescence images and fluorescence quantification of ARG1, INOS, and MFN1 in mice of the control, SeMet, LPS, and LPS + SeMet groups (*n* = 3), with staining for DAPI (blue), ARG1 (red), and INOS and MFN1 (green) (scale bar, 50 μm). **(F)** RT-PCR analysis of mRNA levels of IL-1β, IL-6, and TNF-α in mice of the control, SeMet, LPS, and LPS + SeMet groups (*n* = 3). **(G)** ELISA detection of IL-1β, IL-6, IL-10, and TGF-β levels in the cell supernatants of the control, SeMet, LPS, and LPS + SeMet groups (*n* = 3).

*In vitro* studies using RAW264.7 macrophages confirmed the safety profile of SeMet, with no cytotoxicity observed at concentrations up to 100 μM (Figure 3A). RT-qPCR analysis revealed that the levels of M1 macrophage markers (CD86 and Inos) were significantly lower and those of M2 macrophage markers (CD163 and ARG1) were significantly higher in the LPS + SeMet group than in the LPS group. Notably, SeMet alone could induce M2 polarization, as evidenced by increased ARG1 expression (Figure 3B). Given the critical role of mitochondrial dynamics in macrophage polarization, we examined mitochondrial fusion proteins (Agapito Fonseca et al., 2020; Zarjou et al., 2011). SeMet treatment restored LPS-induced downregulation of Mfn1 expression (Figure 3C), whereas Western blot and immunofluorescence analyses confirm increased ARG1 and Mfn1 protein levels in SeMet-treated groups (Figures 3D,E). Molecular analyses demonstrated that SeMet significantly reduced LPS-induced expression of pro-inflammatory cytokines (IL-1β, IL-6, and TNF-α) at the mRNA level (Figure 3F). ELISA confirmed these findings, showing decreased IL-1β/IL-6 and increased IL-10/TGF-β in cell supernatants (Figure 3G). These findings suggest that SeMet promotes M2 polarization and alleviates inflammatory responses through mitochondrial fusion.

### 3.4 SeMet restores mitochondrial function and inhibits NF-κB signaling

**FIGURE 4:**
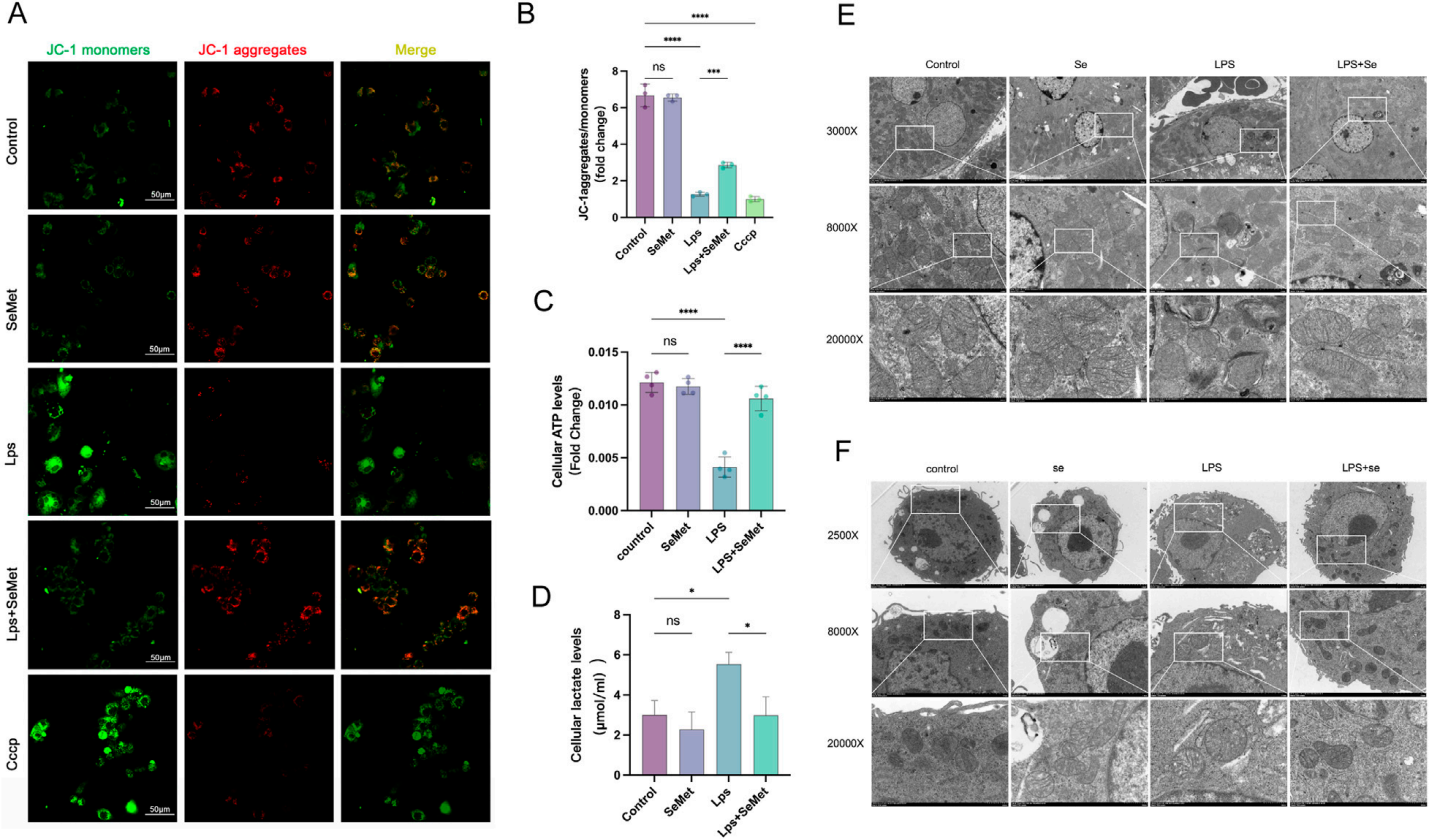
SeMet-induced mitochondrial dynamics improve mitochondrial energy metabolism in RAW264.7 cells. **(A,B)** Representative immunofluorescence images and fluorescence quantification of JC-1 in the cells of the control, SeMet, LPS, LPS + SeMet, and CCCP groups (*n* = 3), with staining for J-aggregates (red) and J-monomers (green) (scale bar, 50 μm). **(C,D)** ATP and lactate levels in the cells of the control, SeMet, LPS, and LPS + SeMet groups (*n* = 3). **(E,F)** Transmission electron microscopy images of kidneys **(E)** and RAW264.7 cells **(F)** from the control, SeMet, LPS, and LPS + SeMet groups at different magnifications: ×3,000, ×8,000, and ×20,000 from top to bottom (scale bars: 5 μm, 2 μm, and 500 nm) (*n* = 3).

**FIGURE 5:**
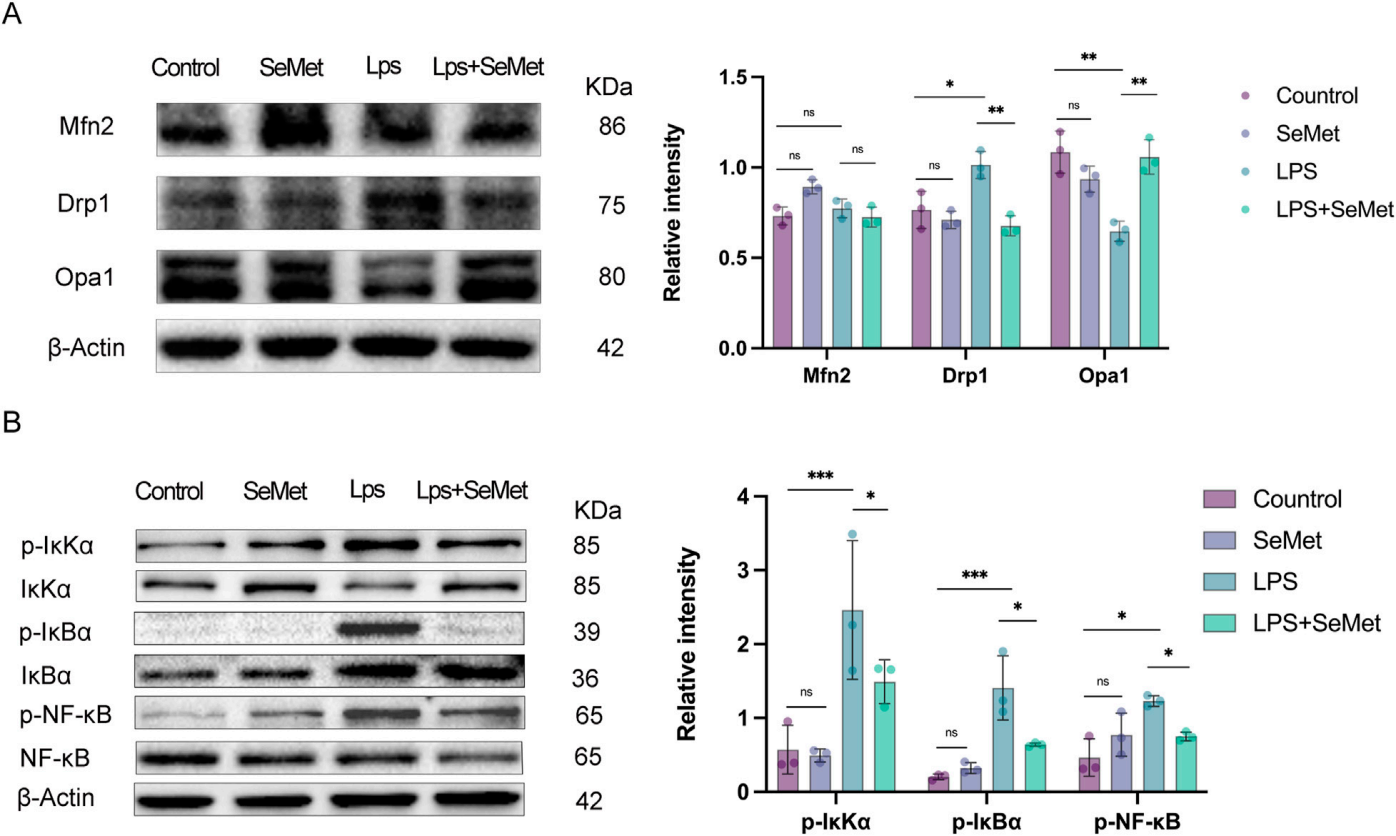
SeMet-induced alterations in mitochondrial dynamics inhibit inflammation by targeting the NF-κB pathway. **(A)** Representative Western blots of Mfn2, Drp1, and Opa1, along with densitometric analysis, in the cells of the control, SeMet, LPS, and LPS + SeMet groups (*n* = 3). **(B)** Representative Western blots of IκKα, P-IκKα, IκBα, p-I κBα, NF-KB, and P-NF-Kb, along with densitometric analysis, in the cells of the control, SeMet, LPS, and LPS + SeMet groups (*n* = 3).

JC-1 staining revealed that SeMet treatment restored mitochondrial membrane potential (ΔΨm), with increased J-aggregates (red) indicating improved mitochondrial function (Figures 4A,B). Metabolic assays showed that SeMet reversed LPS-induced ATP depletion and lactate accumulation (Figures 4C,D), consistent with the lactate and ATP results observed in animal experiments. TEM analysis demonstrated that SeMet ameliorated LPS-induced mitochondrial damage, preserving cristae structure and preventing matrix vacuolization in both renal tissues and cultured macrophages (Figures 4E,F). Mechanistically, we found that SeMet can improve the mitochondrial membrane potential and alleviate mitochondrial ultrastructural damage. We then examined protein levels of mitochondrial dynamics-related markers. LPS treatment resulted in reduced expression of fusion proteins Mfn2 and Opa1 and increased expression of the fission protein Drp1. Following SeMet intervention, Opa1 expression was increased and Drp1 expression returned to normal levels, whereas Mfn2 expression remained unchanged (Figure 5A). Previous studies have shown that Drp1 knockdown or Mfn1 overexpression can reduce NF-κB signaling (Huang et al., 2016). Therefore, we further investigated the effects of SeMet on the NF-κB signaling pathway. As shown in Figure 5B, SeMet significantly reduced LPS-induced phosphorylation of IκKα, IκBα, and NF-κB. These results suggest that SeMet inhibits LPS-induced inflammatory responses by inhibiting the IκKα/IκBα/NF-κB signaling pathway through mitochondrial kinetic regulation and promoting macrophage polarization toward the M2 phenotype.

## 4 Discussion

In this study, we explored the role of SeMet in the LPS-induced SA-AKI mouse model. First, we found that SeMet attenuated LPS-induced renal injury. Subsequently, both *in vitro* and *in vivo* assays showed that SeMet induced the transition from M1 to M2 macrophages in the inflammatory milieu. Furthermore, we found that SeMet attenuates SA-AKI inflammatory responses by modulating altered mitochondrial dynamics to directly promote macrophage polarization toward the M2 phenotype. Additionally, SeMet-mediated modulation of mitochondrial dynamics inhibits the NF-κB pathway, further promoting macrophage polarization toward the M2 phenotype. Collectively, these results provide strong evidence supporting the potential therapeutic application of SeMet in SA-AKI.

SA-AKI is a prevalent complication among critically ill patients, with its pathogenesis closely linked to inflammatory responses (Zarbock et al., 2014). Xia et al. demonstrated that suppressing inflammation can ameliorate sepsis-induced acute kidney injury (Xia et al., 2019; Fan et al., 2022; Zhao et al., 2016). SeMet, a naturally occurring organic selenium compound, has garnered attention for its potent anti-inflammatory and antioxidant properties. For instance, Chen et al. reported that SeMet alleviated LPS-induced inflammation in the eggshell gland by modulating the Keap1/Nrf2/HO-1 pathway (Chen D. et al., 2024). Clinical studies also highlighted the benefits of selenium supplementation in sepsis management. A multicenter trial involving 249 patients with severe systemic inflammatory response syndrome, sepsis, or septic shock revealed that adjunctive sodium selenite treatment reduced morbidity and mortality (Angstwurm et al., 2007). Similarly, Rinaldi et al. observed improved clinical outcomes in patients with infections and organ failure following selenium intervention (Rinaldi et al., 2009).

These findings suggest that SeMet may offer therapeutic advantages for SA-AKI patients, owing to its anti-inflammatory and antioxidant effects. However, the precise mechanisms underlying its role in SA-AKI remain incompletely understood, and its clinical application in sepsis treatment remains limited. In our study, SeMet administration significantly reduced mortality, serum creatinine, and blood urea nitrogen levels. Moreover, it alleviated acute renal dysfunction, pathological renal damage, oxidative stress, inflammatory cytokine release, and apoptosis. Our results provide compelling evidence that SeMet mitigates inflammatory responses and attenuates SA-AKI progression. Wang et al., Chen et al., and Wang et al. demonstrated that shifting macrophage polarization toward the M2 phenotype ameliorates sepsis-induced multi-organ damage (Wang W. et al., 2024; Chen et al., 2023; Wang et al., 2025). Macrophage polarization plays a pivotal role in inflammation, with M1 macrophages driving pro-inflammatory responses and M2 macrophages promoting tissue repair and anti-inflammatory effects (Xie et al., 2020). Our findings align with these reports, further supporting the notion that M2 polarization reduces mortality and dampens inflammatory responses in SA-AKI.

Inflammatory and immune responses are closely related to the cellular metabolism. M1 macrophages primarily rely on glycolysis to meet their rapid energy demands during activation (Mills et al., 2016; Toller-Kawahisa et al., 2025; Zhang J. et al., 2023), whereas M2 macrophages exhibit enhanced OXPHOS (Hamers and Pillai, 2018; Lundahl et al., 2022). LPS upregulates inducible nitric oxide synthase (iNOS) and nitric oxide (NO) production, thereby inhibiting OXPHOS (Bailey et al., 2019; Cyr et al., 2019). In our study, LPS treatment markedly decreased intracellular ATP levels and increased lactate production. However, SeMet intervention reversed these effects, restored mitochondrial membrane potential, and promoted M2 polarization. Additionally, SeMet improved mitochondrial structural integrity in both renal tissues and cellular models of SA-AKI. These observations suggest that SeMet may facilitate M2 polarization by reprogramming macrophage energy metabolism in response to LPS stimulation. Bonnay et al. highlighted the role of mitochondrial fusion in enhancing OXPHOS capacity and facilitating the redistribution of mtDNA between damaged and healthy mitochondria (Bonnay et al., 2020). Impaired mitochondrial fusion can lead to reduced ATP production and increased ROS generation, exacerbating mitochondrial dysfunction (Ryu et al., 2024; Wei et al., 2024). Based on these findings, we hypothesized that SeMet promotes M2 polarization and suppresses inflammatory responses by modulating mitochondrial dynamics. Indeed, SeMet treatment reversed the LPS-induced downregulation of mitochondrial fusion proteins (Mfn1 and Opa1) and attenuated the upregulation of the fission protein Drp1. Enhanced mitochondrial fusion may thus underpin the anti-inflammatory effects of SeMet by promoting OXPHOS and M2 polarization.

The NF-κB pathway is a critical regulator of inflammation. Previous studies have shown that Drp1 knockdown or Mfn1 overexpression reduces NF-κB signaling (Huang et al., 2016). Zhan et al. (2016) further demonstrated that Drp1-mediated mitochondrial fragmentation activates the NF-κB pathway. Consistent with these reports, Shi et al., Zhang et al., and Cao et al. found that SeMet inhibits NF-κB to alleviate inflammation (Shi et al., 2020; Zhang Z. et al., 2023; Cao et al., 2023). In our study, SeMet modulated mitochondrial dynamics and suppressed NF-κB pathway activation. This was accompanied by reduced levels of pro-inflammatory cytokines (IL-1β, IL-6, and TNF-α) in the serum and cell supernatants of SA-AKI mice. These results align with the findings of Shen et al. and Zhang et al., who reported that SeMet inhibits NF-κB to suppress inflammation (Shen et al., 2015; Zhang Z. et al., 2023). Furthermore, Luo et al. demonstrated that NF-κB inactivation promotes M2 polarization (Luo et al., 2022), supporting our conclusion that SeMet modulates mitochondrial dynamics to inhibit NF-κB, thereby driving M2 polarization and attenuating SA-AKI-associated inflammation.

Although, in this study, we elucidate the potential mechanisms by which SeMet alleviates SA-AKI inflammation through mitochondrial dynamics and macrophage polarization, several limitations warrant acknowledgment. First, the causal relationship between mitochondrial dynamics and the observed effects remains to be directly validated. Second, our analysis relied on total kidney tissue protein, which precludes cell-type-specific insights. Third, the upstream mechanisms by which SeMet regulates mitochondrial dynamics remain unclear. Fourth, LPS, an endotoxin derived from Gram-negative bacteria, rapidly induces a robust systemic inflammatory response, making it a widely utilized tool for modeling sepsis due to its operational simplicity and high reproducibility (Su et al., 2017). Although the cecal ligation and puncture (CLP) model more closely mimics the clinical features of sepsis, its technical complexity, significant inter-individual variability, and sensitivity to procedural variables (e.g., puncture size and ligation site) compromise its consistency and reproducibility (Su et al., 2024; Dejager et al., 2011; Ruiz et al., 2016; Alverdy et al., 2020; Aydın and Bekmez, 2023). Given these limitations, the LPS-induced model was selected for this study to investigate the role of inflammatory responses in SA-AKI pathogenesis and to evaluate potential therapeutic interventions. Future studies should incorporate genetic interventions, single-cell technologies, and more clinically relevant models (e.g., CLP) to further elucidate these mechanisms and explore the translational potential of SeMet in SA-AKI treatment.

## 5 Conclusion

**FIGURE 6:**
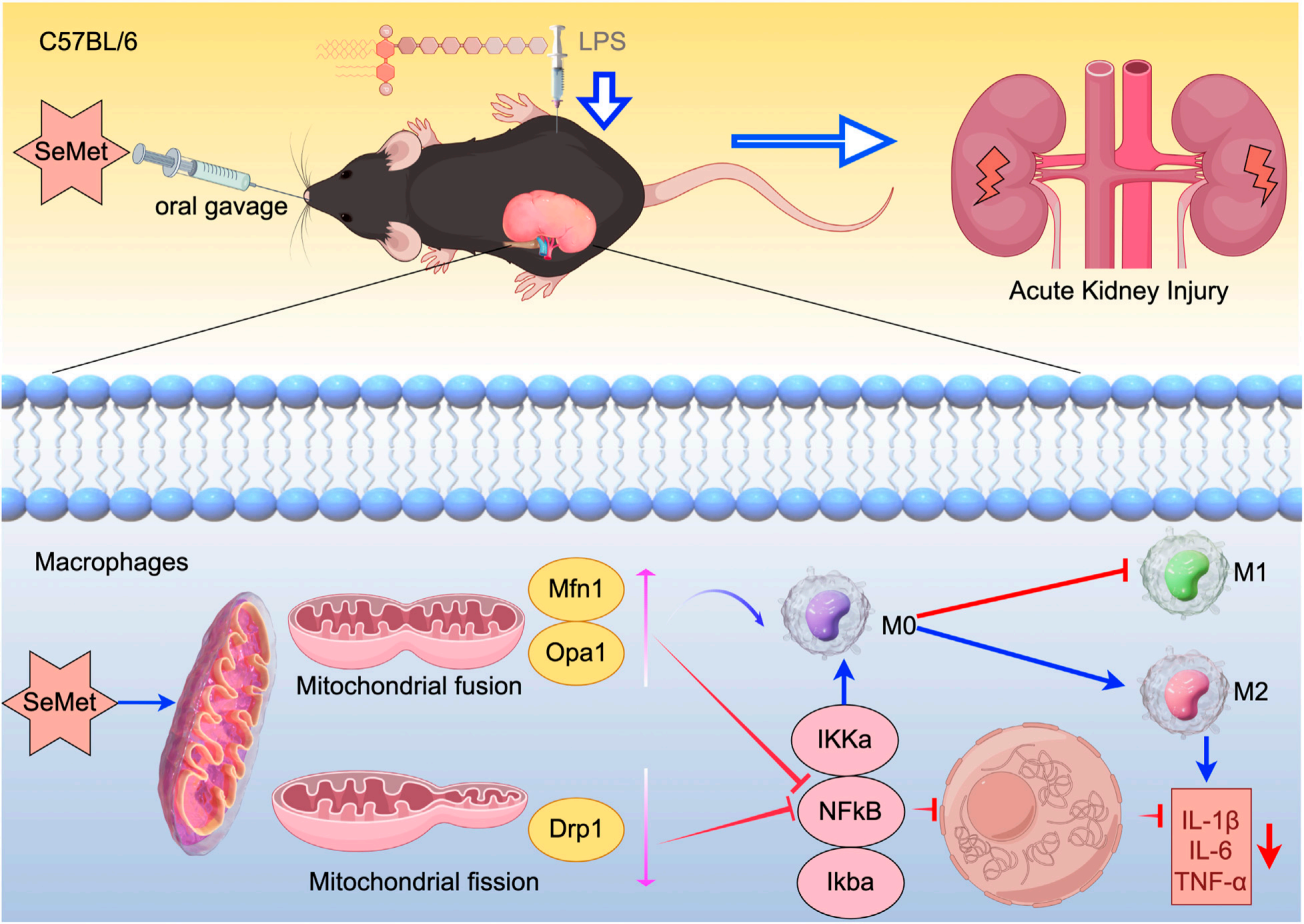
Schematic diagram of the mechanism: SeMet attenuates inflammatory responses by modulating altered mitochondrial dynamics and targeting inhibition of the NF-κB signaling pathway to promote macrophage polarization toward the M2 phenotype.

In this study, we demonstrated that SeMet promotes M2 macrophage polarization by modulating mitochondrial dynamics and inhibiting the NF-κB pathway, thereby exerting anti-inflammatory effects in LPS-induced RAW264.7 cells and a murine SA-AKI model. Enhanced mitochondrial fusion further facilitated metabolic reprogramming toward the M2 phenotype (Figure 6). These findings highlight SeMet as a promising therapeutic candidate for SA-AKI.

## Data Availability

The original contributions presented in the study are included in the article/Supplementary Material; further inquiries can be directed to the corresponding authors.

## Abbreviations

AKI, acute kidney injury; LPS, lipopolysaccharide; SA-AKI, sepsis-associated acute kidney injury; SeMet, selenomethionine; Mfn1/2, mitochondrial fusion protein-1/2; Opa1, optic nerve atrophy 1; Drp1, dynamin-related protein 1; M1, M1 macrophages; M2, M2 macrophages; ROS, reactive oxygen species; OXPHOS, oxidative phosphorylation; NF-κB, nuclear factor-kappa B; ATP, adenosine triphosphate.
