# Supplementary material for: Selenomethionine alleviates LPS-induced septic kidney injury by regulating mitochondrial dynamics changes
Source: Front Pharmacol. 2025 Sep 8;16:1606365. doi: 10.3389/fphar.2025.1606365 (PMC12450938; doi:10.3389/fphar.2025.1606365)
Supplement: Supplementary file 1 [file Table1.docx]

Supplementary Material

# Supplementary Table 1

Primer List

| genetics | orientations | sequences (5'-3') |
| --- | --- | --- |
| IL-1β | F | TGAAGAAGAGCCCATCCTCTG |
|  | R | CTTGTTGGTTGATATTCTGTCC |
| IL-6 | F | AAGAGTTGTGCAATGGCAATTC |
|  | R | CATGTACTCCAGGTAGCTATGG |
| TNF-α | F | GCCTCCCTCTCATCAGTTCTA |
|  | R | GGCAGCCTTGTCCCTTG |
| CD80 | F | GGCCCTCCTCCTTGTGATG |
|  | R | CTGGGCCTGCTAGGCTGAT |
| iNOS | F | CAAGCACATTTGGGAATGGAGA |
|  | R | CAGAACTGAGGGTACATGCTGGAG |
| CD163 | F | AGGGACCTGGATGGATGACA |
|  | R | TGTACCGCACCCTCCATCTA |
| Arg-1 | F | AGCTCTGGGAATCTGCATGG |
|  | R | ATGTACACGATGTCTTTGGCAGATA |
| Mfn1 | F | CCTACTGCTCCTTCTAACCCA |
|  | R | AGGGACGCCAATCCTGTGA |
